# Metallothionein and Superoxide Dismutase—Antioxidative Protein Status in Fullerene-Doxorubicin Delivery to MCF-7 Human Breast Cancer Cells

**DOI:** 10.3390/ijms19103253

**Published:** 2018-10-20

**Authors:** Marta Kepinska, Rene Kizek, Halina Milnerowicz

**Affiliations:** 1 Department of Biomedical and Environmental Analyses, Faculty of Pharmacy with Division of Laboratory Medicine, Wroclaw Medical University, Borowska 211, 50-556 Wroclaw, Poland; kizek@sci.muni.cz (R.K.); halina.milnerowicz@umed.wroc.pl (H.M.); 2 Department of Human Pharmacology and Toxicology, Faculty of Pharmacy, University of Veterinary and Pharmaceutical Sciences Brno, Palackeho nam. 1949, 612 42 Brno, Czech Republic

**Keywords:** breast tumors, doxorubicin, drug delivery systems, fullerene, nanoparticles, metallothionein, superoxide dismutase

## Abstract

Doxorubicin (DOX) is one of the most frequently used anticancer drugs in breast cancer treatment. However, clinical applications of DOX are restricted, largely due to the fact that its action disturbs the pro/antioxidant balance in both cancerous and non-cancerous cells. The aim of this study was to investigate the influence of fullerene (C_60_) in cell treatment by DOX on the proliferation of human breast cancer cells (MCF-7), concentration of metallothionein (MT) and superoxide dismutase (SOD), and SOD activity in these cells. The use of C_60_ in complexes with DOX causes a change in the level of cell proliferation of about 5% more than when caused by DOX alone (from 60–65% to 70%). The use of C_60_ as a DOX nanotransporter reduced the MT level increase induced by DOX. C_60_ alone caused an increase of SOD1 concentration. On the other hand, it led to a decrease of SOD activity. C_60_ in complex with DOX caused a decrease of the DOX-induced SOD activity level. Exposure of MCF-7 cells to DOX-C_60_ complexes results in a decrease in viable cells and may become a new therapeutic approach to breast cancer. The effects of C_60_ in complexes with DOX on MCF-7 cells included a decreased enzymatic (SOD activity) and nonenzymatic (MT) antioxidant status, thus indicating their prooxidant role in MCF-7 cells.

## 1. Introduction

Breast cancer is the most commonly diagnosed cancer in women worldwide and also the leading cause of mortality in American women [1]. Elevated reactive oxygen species (ROS) levels are found in the majority of cancer cells [2]. Free radicals, operating on a variety of signal pathways, increase the expression of proteins responsible for increasing the number of cell divisions [3]. This action results in increased cell proliferation and tumor mass growth [4].

Anthracyclines (comprising doxorubicin) are regularly used in breast cancer treatment [5,6]. Doxorubicin (DOX) is a DNA interchelator which inhibits topoisomerase II, thereby inhibiting cancer cell growth. DOX can be converted by reductases to anthracycline semiquinone free radicals. In aerobic conditions, they are able to reduce molecular oxygen to O_2_^−^ and H_2_O_2_. The most severe negative effect of anthracyclines is, therefore, cardiomyopathy leading to congestive heart failure, which can also be caused by increased oxidative stress. One strategy to mitigate the side effects of DOX is the use of drug delivery systems [7].

Nanoparticles can be used for targeted drug delivery and controlled release [8]. It has been found that fullerene-DOX therapy is as effective as therapy with the drug itself; however, no characteristic toxic effects have been observed in the case of complexed DOX [9]. Fullerenes (C_60_) have the ability to accumulate in the tumor mass. This property is attributed to the small size of these carbon molecules, thanks to which they may use the so-called enhanced permeability and retention effect (EPR), which means that they penetrate easily through the less-tight blood vessels nourishing the cancer [10]. The use of a nanotransporter makes it possible to regulate the release of the coupled drug, breaking the drug-transporter binding at a suitable pH, e.g., a cancer cell that is characteristic of the environment [11]. These actions ensure a selective accumulation of the drug in the tumor and significantly reduce the amount necessary to obtain a therapeutic effect. C_60_, due to its antioxidant and radical scavenging activity, has the potential to mitigate the DOX side effects triggered by ROS. It is believed that the ability of fullerenes to scavenge free radicals is the reason why their administration to cancer cells reduces the concentration of ROS in the cell, thus inhibiting the activation of proto-oncogenes, tumor growth and angiogenesis [12]. The antioxidant defense system against intracellular levels of ROS is composed of non-enzymatic molecules, such as metallothionein, and of antioxidant enzymes, such as superoxide dismutase [13]. Antioxidant capability is one of the main functions of metallothioneins (MTs), a group of low-molecular cysteine-rich metalloproteins [14,15]. In breast cancer, MT overexpression has shown to be predominantly associated with poor prognosis in spite of having an antioxidant role. MTs influence tumor growth and promote cell proliferation and cellular repair processes, enhancing resistance to chemotherapy and preventing apoptosis [16,17]. The second of the main defense mechanisms against ROS is the presence of ROS decaying enzymes called superoxide dismutases (SODs). Their action is based on the enzymatic reduction of oxygen radicals to the less toxic hydrogen peroxide, which is then decomposed by appropriate catalases. SOD1, a copper-zinc (Cu/Zn SOD) isoform, is a variant located in the cellular cytoplasm and mitochondrial inter-membrane space. It is overexpressed in cancers and its activity may be essential to maintaining cellular ROS under the critical threshold [18]. SOD1 is overexpressed in malignant breast cancer cells (MCF-7). Conversely, it is reduced in the non-tumorigenic MCF10A cell line [18]. In breast cancer cells, intracellular H_2_O_2_ levels are found to be higher due to the altered SOD expression and activity in addition to a decreased expression of catalase [2]. For this reason, it is important to know the effect of applied therapies on the activity of SOD in the cells. It can be of particular importance for using such drugs as DOX, which has serious side effects associated with ROS formation.

The goals of the present study were to determine the level of the MT-1/2 and SOD1 as well as Zn/Cu SOD activity in the MCF-7 cell line when the cells were exposed to DOX alone and in complexes with C_60_ as nanotransporters and antioxidants selected to protect the body from the effects of DOX therapy.

## 2. Results

### 2.1. Biophysical Characterization of C_60_–DOX (Fullerene-Doxorubicin) Complexes

**Figure 1:**
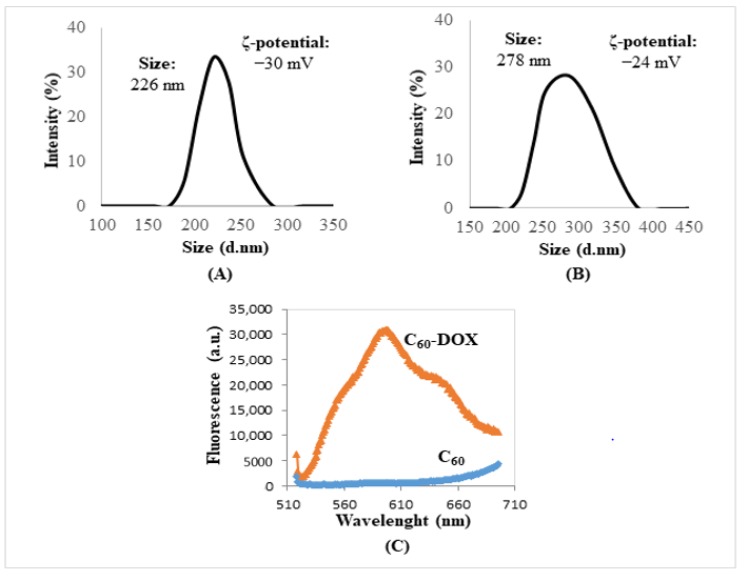
Biophysical characterization of the fullerene (C_60_) and complexes of C_60_–doxorubicin (DOX)**.** Size and zeta potential of the C_60_ (**A**) and C_60_–DOX complexes (**B**). (**C**) Fluorescence spectra of C_60_–DOX complexes (concentration of fullerene: 25 mg/mL)—measured immediately after their preparation without further specimen processing. For other experimental conditions, see Material and Methods. ζ—zeta; C_60_—fullerene; DOX—doxorubicin.

C_60_–DOX complexes were analyzed by biophysical methods. The morphology of C_60_–DOX conjugates was observed through a scanning electron microscope and aggregates of C_60_, and C_60_ in complex with DOX were observed. Structures of C_60_–DOX complexes of about 100 nm to several microns in diameter at 150,000× magnification were observed. The hydrodynamic diameter of C_60_, analyzed by dynamic light scattering, was 226 nm (Figure 1A). The size of C_60_ in complex with DOX increased to 278 nm (Figure 1B). A zeta (ζ) potential change of 6 mV, from ‒30 mV (C_60_) to ‒24 mV (C_60_–DOX complexes) was observed. Fluorescence of C_60_–DOX complexes was measured after washing off free DOX (Figure 1C). Concentrations of DOX in C_60_–DOX complexes measured by fluorescence intensity were calculated from the calibration curve of the fluorescence signal of free DOX, obtaining 1005 and 1998 nM of DOX bounded in C_60_–DOX complexes. The drug entrapment efficiency (EE) of oxidized C_60_–DOX was 78–80%.

### 2.2. Proliferation Status of MCF-7 Human Breast Cancer Cells Treated by C_60_–DOX Complexes

**Figure 2:**
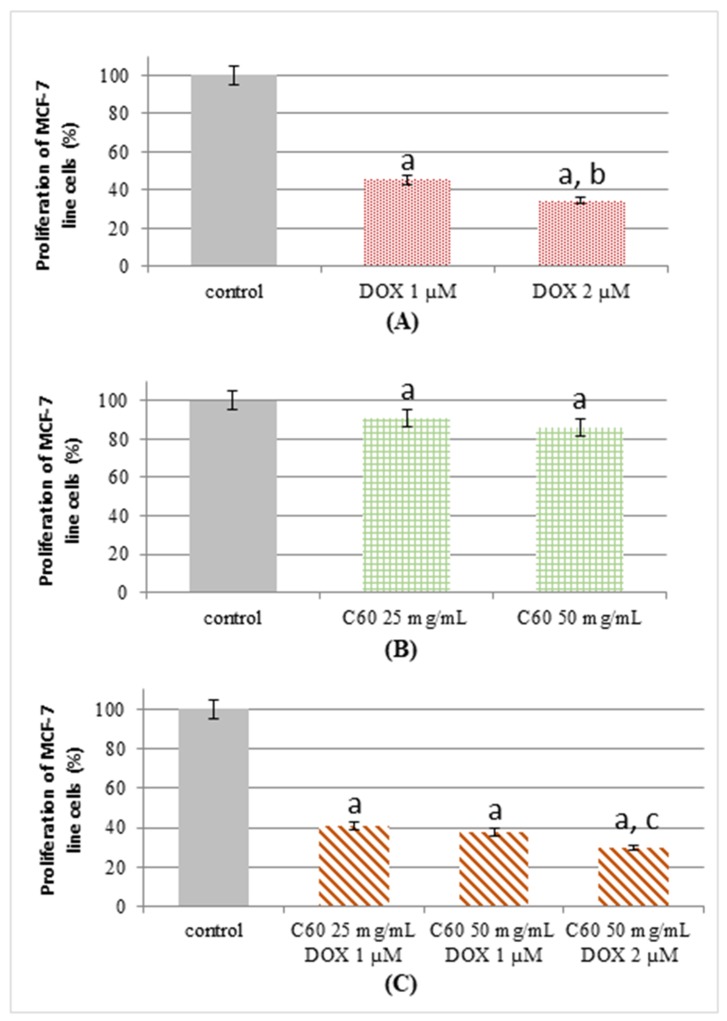
Proliferation of MCF-7 cells. Proliferation of MCF-7 cells treated by (**A**) DOX, (**B**) C_60_, (**C**) complexes of C_60_–DOX. For other experimental conditions, see Material and Methods. ^a^ *p* < 0.05 when compared to control cells; ^b^ *p* < 0.05 when compared to cells treated by 1 µM DOX; ^c^ *p* < 0.05 when compared to cells treated by C_60_–DOX complexes (25 mg/mL C_60_-1 µM DOX and 50 mg/mL C_60_-1 µM DOX).

The current experiments were designed to characterize the influence of C_60_ on cellular proliferation in DOX treatment. We have found a decreased proliferative activity of MCF-7 cells treated by DOX compared to control (Figure 2A). C_60_ alone caused a decrease of MCF-7 cell proliferation by about 10–15% (Figure 2B); however, DOX influenced cell proliferation to a much greater extent, causing a decrease of cell proliferation by 60–65%. The results obtained after the incubation of MCF-7 cells with the C_60_–DOX complexes indicate a decrease of cell proliferation when compared with the effect of DOX alone. C_60_–DOX complexes showed a 65–70% lower proliferation of breast cancer cells compared to the control and when higher concentration of C_60_ (50 mg/mL) was used, the difference was statistically significant. This indicates that, besides the effect of the nanocarrier-loaded anticancer drug, the lower proliferation activity of MCF-7 cancer cells was also a consequence of C_60_ presence. Figure 2C indicates that the C_60_–DOX combination is more toxic for tumor cell lines than free DOX.

### 2.3. The Effect of Complexes of C_60_–Doxorubicin on Metallothionein Concentration

**Figure 3:**
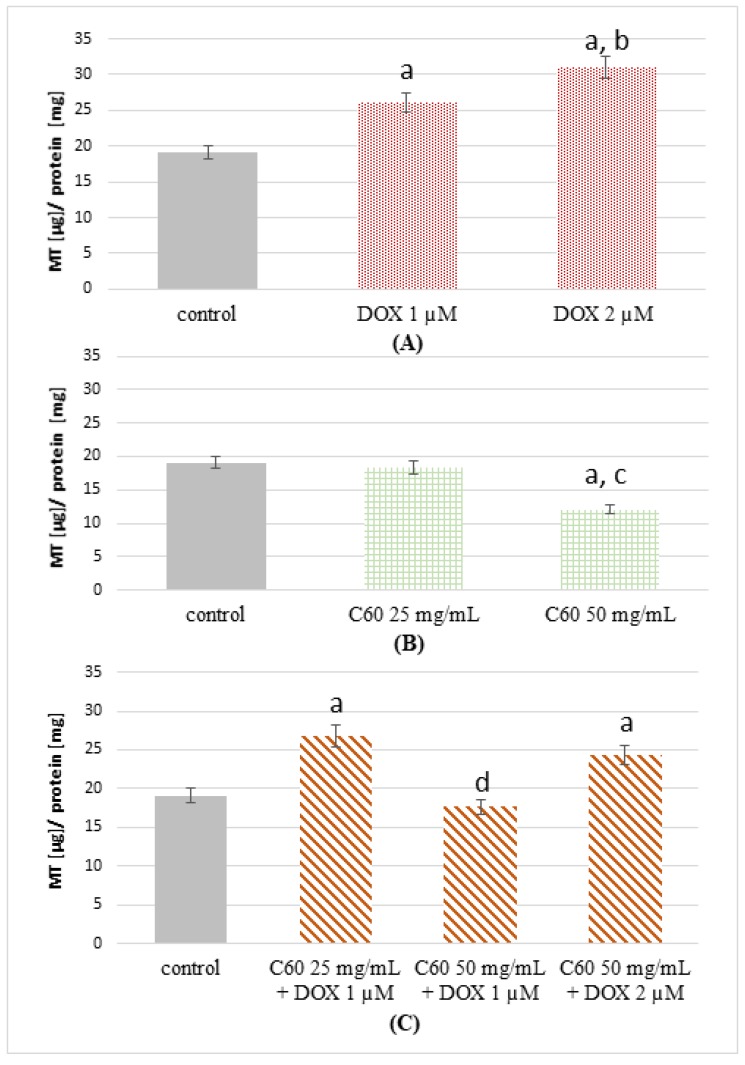
Concentration of metallothionein (MT) in MCF-7 cells treated by DOX, C_60_ and C_60_–DOX complexes. Concentration of MT-1/2 in MCF-7 cells treated by (**A**) DOX, (**B**) C_60_, (**C**) complexes of C_60_–DOX. For other experimental conditions, see Material and Methods. ^a^ *p* < 0.05 when compared to control cells; ^b^ *p* < 0.05 when compared to cells treated by 1 µM DOX; ^c^ *p* < 0.05 when compared to cells treated by 25 mg/mL C_60_; ^d^ *p* < 0.05 when compared to cells treated by C_60_–DOX complexes (25 mg/mL C_60_-1 µM DOX and 50 mg/mL C_60_-2 µM DOX).

The concentration of MT-1/2 in cells treated with 1 μM and 2 μM of DOX was examined. A higher concentration of MT-1/2 in the lysate of cells treated with DOX was found in comparison with the control (Figure 3A).

The increase was 1.4-fold when 1 μM of DOX was used, and a 1.6-fold increase in MT-1/2 concentration was noted for cells treated with 2 μM of DOX compared to control. Cells treated with 2 μM DOX were characterized by 1.15 times higher concentration of MT-1/2 compared to cells treated with 1 μM DOX. Conversely, in MCF-7 cells treated by 25 mg/mL C_60_, a statistically insignificant decrease of MT-1/2 concentration was observed (Figure 3B). A much lower concentration of MT-1/2 was found in the case of cells treated by 50 mg/mL of C_60_. They were 1.6-fold lower than in the case of the control. In the cells treated by C_60_–DOX complexes (25 mg/mL-1 μM and 50 mg/mL-2 μM, respectively), the determined MT-1/2 concentration was higher than in the control (Figure 3C). However, in the samples treated by C_60_–DOX (50 mg/mL-1 μM), the MT-1/2 concentration was almost the same as in the control. Therefore, the influence of C_60_ use in DOX action in human breast cancer MCF-7 cells is characterized by changes in the expression of MT involved in the control of the oxidative status in the cell.

### 2.4. Influence of C_60_ on the Concentration and Activity of SOD (Superoxide Dismutase) in MCF-7 Treatment by DOX

**Figure 4:**
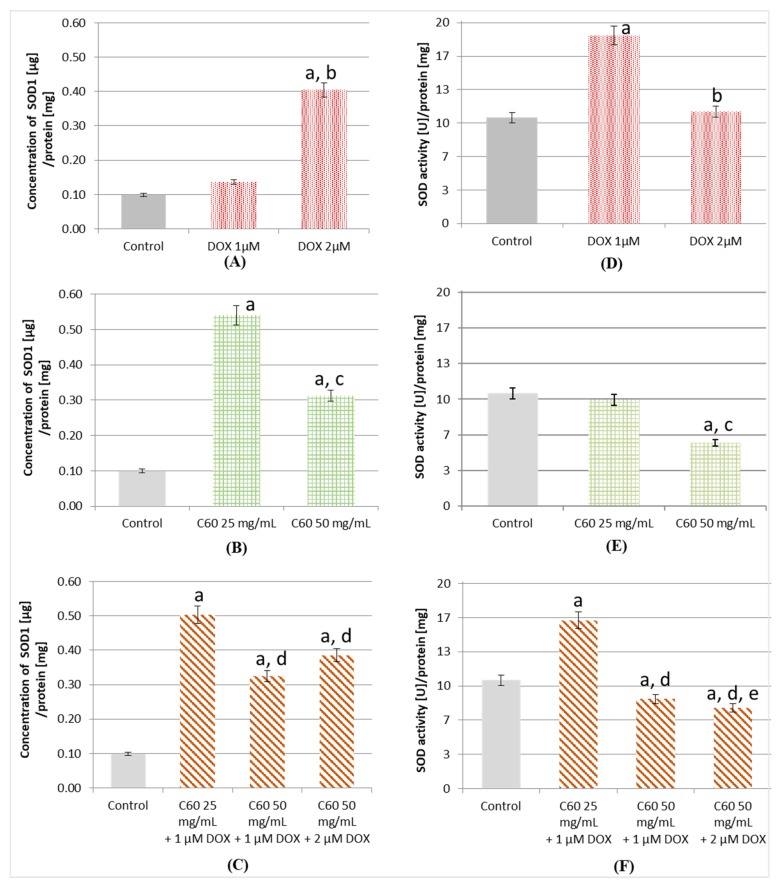
Concentration of superoxide dismutase 1 (SOD1) and activity of SOD in MCF-7 cells treated by DOX, C_60_ and C_60_–DOX complexes. Concentration of SOD1 in MCF-7 cells treated by (**A**) DOX, (**B**) C_60_, (**C**) complexes of C_60_–DOX. Activity of SOD in MCF-7 cells treated by (**D**) DOX, (**E**) C_60_, (**F**) complexes of C_60_–DOX. C_60_—fullerene, DOX—doxorubicin. For other experimental conditions, see Material and Methods. ^a^ *p* < 0.05 when compared to control cells; ^b^ *p* < 0.05 when compared to cells treated by 1 µM DOX; ^c^ *p* < 0.05 when compared to cells treated by 25 mg/mL C_60_; ^d^ *p* < 0.05 when compared to cells treated by C_60_–DOX complexes (25 mg/mL C_60_-1 µM DOX); ^e^ *p* < 0.05 when compared to cells treated by C_60_–DOX complexes (50 mg/mL C_60_-1 µM DOX).

The concentration of SOD1 in cells treated with different concentrations of DOX was examined. A higher concentration of SOD1 was found in samples containing a lysate of MCF-7 cells exposed to DOX than in the control (Figure 4A). When 1 μM of DOX was used, there was an increase of about 36%; more than a fourfold increase in SOD1 concentration was noted for cells treated with 2 μM of DOX compared to control. Cells treated with 2 μM DOX were characterized by almost three times higher concentration of SOD1 compared to cells treated with 1 μM DOX. Among the MCF-7 cells treated by C_60_ only, a higher concentration of SOD1 was found in the case of treatment by 25 mg/mL than by 50 mg/mL of C_60_ (Figure 4B). It was almost twice as high as in the case of 50 mg/mL C_60_ treatment. In both cases (25 and 50 mg/mL), the concentrations of SOD1 in the cells were higher than in the control. Treatment by C_60_–DOX complexes showed higher concentrations of SOD1 compared to the untreated MCF-7 cells (Figure 4C). In the case of C_60_–DOX application (25 mg/mL, 1 μM), the determined SOD1 concentration was almost the same as in the samples treated by 25 mg/mL C_60_, whereas in the samples treated by C_60_–DOX (50 mg/mL, 2 μM), the SOD1 concentration was higher than in the case of C_60_ 50 mg/mL treatment and almost the same as the concentration observed in the cells treated by DOX 2 μM. The samples treated using C_60_ (25 mg/mL) and the complex of C_60_ (25 mg/mL) with DOX (1 μM) had the highest SOD1 concentration—almost five times higher than observed in the control.

SOD activity was analyzed in lysates of MCF-7 cells treated with various concentrations of DOX (1 and 2 μM). The obtained results showed almost two times higher SOD activity for cells treated with 1 μM DOX compared to control (Figure 4D). In the case of cells treated with 2 μM DOX, the activity of SOD was approximately 5% higher than that observed in the control. Conversely, in MCF-7 cells treated by 25 mg/mL C_60_, a statistically insignificant decrease of SOD activity was observed (Figure 4E) and a much lower activity of SOD was found in the case of cells treated by 50 mg/mL of C_60_.

The highest reported SOD activity in cells treated with complexes was recorded for 25 mg/mL C_60_ with 1 μM DOX (16.23 U/mg protein). It was noticeably higher than the activity found in the control (10.44 U/mg protein). In the rest of the samples, the measured values were smaller than those observed in the MCF-7 control. Cells treated with the C_60_–DOX complex (50 mg/mL C_60_ with 1 μM DOX or with 2 μM DOX) were characterized by higher SOD activity (8.65 and 7.7 U/mg protein, respectively) as compared to cells treated by 50 mg/mL C_60_ only (5.85 U/mg protein). Each of the examined complexes had a SOD1 concentration greater than the control samples and at the same time, with the exception of cells treated by DOX (1 and 2 μM) and C_60_–DOX complexes (25 mg/mL, 1 μM), SOD activity was lower than that observed in the control.

Concentrations of SOD1 in every analyzed MCF-7 cell lysate were higher than in control, but SOD activity was smaller, or its increase was lower, than the increase of concentration, except for cells treated by 1 μM DOX. The activity of SOD in conversion to SOD1 concentration in MCF-7 cells treated by DOX, C_60_ and C_60_–DOX complexes was calculated (Table 1). The obtained results indicate that SOD activity per SOD1 concentration was reduced compared to controls, except for cells treated by 1 μM DOX. The lowest proportion of SOD activity/SOD concentration was found in the cell lysate treated by C_60_.

## 3. Discussion

ROS, which are responsible for the side effects of anthracyclines, are generated as products of an electron transfer between a quinone moiety of anthracyclines and oxygen and other donor molecules [19]. This was confirmed during studies on the effect of antioxidants, the antioxidant properties of which caused a decrease in the cytotoxic effect of DOX [20]. It has been demonstrated on the example of breast cancer cells, that short-term high ROS concentration has a therapeutic effect, whereas in the case of chronic high ROS concentration, the proliferation of the examined cells was significantly increased [1]. MCF-7 breast cancer cells in which the level of ROS was experimentally increased were characterized by greater motility compared to controls and were characterized by a more invasive nature [21].

Fullerenes (C_60_), due to their special physico-chemical properties, make great candidates for drug transporters [22]. The difference in their size measured by scanning electron microscopy and by dynamic light scattering was discussed in our previous work and obtained data are in agreement with other reports, in which the fullerene size is between 150−490 nm [11]. The structure of C_60_ enabling the adoption of up to six electrons indicates the possible antioxidant effect of this form of carbon [23]. These properties caused C_60_ to become an object of analysis to determine their ability as scavengers of free radicals [24]. The ability of C_60_ to reduce and inactivate ROS has been proven in both in vitro and in vivo studies, during which Gharbi et al. compared the harmful effects of tetrachloromethane administered with the addition of C_60_ on the livers of mice [25]. C_60_ may function as a free radical scavenger and it strongly suppresses the toxicity of DOX in animal models [26]. Panchuk et al. reported that the DOX conjugation with C_60_ led to a 1.5- to 2-fold increase in DOX toxicity towards various human tumor cell lines compared with such an effect when the drug is used alone. The increased cytotoxic activity of the C_60_–DOX complex is a result of the cumulative effect of DOX and C_60_ [20]. The special properties of fullerenes are known to cause their accumulation in tumor cells, which is referred to as the enhanced permeability and retention (EPR) effect [27]. This would also mean increased DOX concentration inside the cells as compared to the cells treated by DOX alone. It has been shown that the administration of fullerene derivative Gd@C_82_(OH)_22_ (gadolinium metallofullerenol) significantly impairs the process of angiogenesis in tumor cells, reducing blood perfusion and nutrition of the entire tumor mass [28]. This conjugation with DOX repressed the cancer cell proliferation in vitro by blocking the G2-M cell cycle, leading to apoptosis. In this work, we have shown that C_60_ alone caused a decrease of MCF-7 cell proliferation by about 10%, and DOX (1–2 µM) influenced cell proliferation to a much greater extent, causing a decrease of cell proliferation by 60–65%.

In the present study, the effect of C_60_ on MT and SOD levels in DOX-treated MCF-7 cells was tested. The results obtained could be of clinical significance, therefore DOX concentration was used at a level comparable to that achieved in patients treated by this drug. Generally, plasma concentration of DOX falls into the range of 1–2 µM, with the maximal initial amount of 5 µM DOX and the lowest reported amount of 0.3 µM [29]. In the present study, a clinically relevant concentration of 1–2 µM DOX was used [30].

An increased expression of MT has been shown in various human tumors, including breast, colon, liver, lung etc. Breast cancer was one of the first tumor types studied for the level of MT expression, and an increase of MT-1/2 is associated with poorer prognosis [31]. Elevated MT levels correlate with a high tumor grade, chemoresistance, increased cell proliferation and reduced apoptosis [32]. In breast cancer cells, the downregulation of MT-2A by siRNA results in the induction of growth arrest and apoptosis; however, the exact mechanism by which MT-2A influences cancer cell invasion in breast cancer has not been well explained [33]. MT serves as a zinc storage site with no recognizable direct DNA-binding site, that can induce its biological effects via activation and/or donation of zinc to zinc fingers of transcription factors, next leading to transactivation of the genes [34]. The overexpression of MT stimulated cellular multiplication and exerted effects on the proto-oncogene and tumor suppressor genes [34]. In contrast, the downregulation of MT in MCF-7 cells with an 18-mer antisense phosphorothioate inhibited growth and initiated apoptosis, suggesting a close involvement of the MT-2A isoform in the proliferative activity of breast cancer cells [34,35,36,37]. MTs are known to be inducible by oxidative stress [15,38]. In this work, we have shown that treatment with DOX alone causes an increase in MT concentration. Concentration of MT-1/2 in MCF-7 cells treated with DOX was higher (26 and 31 µg/mg protein for 1 and 2 µM DOX, respectively) than in the control (19 µg/mg protein for the control). Sun et al. have shown that the amount of MT secreted by murine cardiomyocytes increase with prolonged use of DOX. In our studies, we observed a higher MT level in MCF-7 cells caused by DOX. Oxidative stress triggers the mobilization of zinc from MT. This may either constitute a general pathway by which zinc is distributed in the cell or restricted to conditions of stress in which zinc is needed in antioxidant defense systems [39]. MT is a zinc-binding protein, and under oxidative stress conditions, zinc is released from MT [40]. MCF-7 cells treated by C_60_ showed a lower MT-1/2 concentration (18 and 12 µg/mg protein for 25 and 50 mg/mL C_60_, respectively). Application of 50 mg/mL of C_60_ in complex with DOX 1 or 2 µM resulted in a decrease in MT concentration, which increased in MCF-7 cells under the influence of DOX itself. The antioxidant properties of C_60_ probably caused a decrease in MT expression, the secretion of which is stimulated by, among others, oxidative stress. Abdel-Mageed and Agrawal suggested that inhibitors of MT may potentially find therapeutic application in inducing apoptosis in neoplastic cells [34]. Studies conducted by Yin et al. also indicate that DOX causes the induction of MT as well as SOD expression [41].

Increased ROS concentration caused by anthracycline antibiotics, including DOX, is related to their cytotoxic effect [42]. This is important in the context of effective anticancer therapy, since many cancer cells have been shown to overexpress SOD1, which may impair the action of DOX [18]. The results obtained in this study showed an increased concentration of SOD1 in cells treated with DOX, and a higher activity of SOD, from 10.4 U/mg in the control to 18.5 U/mg in the case of 1 μM DOX treatment and to 11.0 U/mg in the case of 2 μM DOX use. A slight increase in the level of SOD activity in MCF-7 cells after the administration of cytostatic drugs (topotecan) has also been demonstrated by Timur et al. (47 to 69 U/mg) [43]. This can be attributed to adaptive changes occurring in cancer cells, aimed at increasing the survival of cells exposed to high concentrations of ROS generated by the drug. In the case of MCF-7 cells, the increased SOD1 concentration was assigned as one of the direct mechanisms conditioning the resistance of tumor cells to DOX action [44]. The discovery of this mechanism has started a new line of research investigating therapy with anthracycline antibiotics. Tocopherols or 1.25 dihydroxyvitamin D3 are noteworthy among the substances tested to increase the susceptibility of MCF-7 cells to DOX [45].

There are no such studies for fullerenes; however, there are reports of blocking the activity of SOD by multi-wall carbon nanotubes [46]. Fullerenes, as stated above, have an oxidative stress reduction ability [25,47]. The results obtained in this study indicate that samples treated with C_60_ show lower SOD activity than the controls, despite the higher SOD1 concentration. This may indicate that the activity of SOD is probably blocked by C_60_. The confirmatory results were obtained in samples containing cells treated with C_60_ -DOX complexes at concentrations of 50 mg/mL C_60_-1 μM DOX and 50 mg/mL C_60_-2 μM DOX, in which the determined activities were smaller than those determined for the control. This may imply the inhibition of SOD activity in tumor cells by C_60_ at a concentration of 50 mg/mL. Despite the higher concentration of SOD1 in almost every MCF-7 cell lysate, the obtained SOD activities were smaller, except for the cells treated with the complex of 25 mg/mL C_60_ with 1 μM DOX and with 1 μM DOX alone. This indicates that SOD1 was present in the samples tested, but its activity was reduced by C_60_ compared to controls.

Proportion of SOD activity per SOD1 concentration was reduced compared to control in all analyzed samples except for cells treated by 1 μM DOX. Treatment of cells by this DOX concentration caused an increase of said ratio by 27% compared to control, which means that the increase in SOD activity was greater than the increase in SOD1 concentration. SOD1 expression may be altered following certain exogenous stimuli. However, the production of a fully formed, active CuZnSOD dimer formation by post-translational processing also provides a means by which SOD activity can be altered in cells. Glutathionylation, palmitoylation, succinylation and acetylation are among the PTMs of SOD1. Lysine 123 acetylation is a reversible PTM that regulates the interaction, subcellular localization, folding and activity of many proteins [48]. Specific conditions, such as H_2_O_2_ and superoxide concentration, appear to favor SOD activity [49]. The maturation of SOD1 into a fully active enzyme requires several steps, including the insertion of Cu and Zn and oxidation of a critical disulfide that is essential for SOD activity [50]. This oxygen-responsive disulfide process is catalyzed by the copper form of copper chaperone for SOD1 and appears to be part of a physiological cycle that regulates the amount of active SOD1 [51]. These findings are important for understanding the molecular mechanism of SOD1 maturation and activation in its defense against toxic superoxide anions [52]. This copper chaperone perform more than just supplies copper; it exhibits both sulfhydryl oxidase and protein disulfide isomerase activities that enable regulation in response to oxidative stress [51].

The lowest proportion of SOD activity/SOD concentration was found in the cell lysate treated by C_60_ alone. In addition to the aforementioned results and the studies concerning the operation of multi-wall carbon nanotubes, there are also reports on the ability of C_60_ to block the center of other active enzymes. Both Zhu et al. and Innocenti et al. used computational techniques to show that fullerene C_60_ derivatives act as enzyme inhibitors. These results concerned 2 different enzymes: carbonic anhydrase and HIV-1 protease, and in both papers, the described principle of fullerenes action was the same. The proposed mechanism in both cases consisted in the fullerene molecule occupying the active site of the enzyme and inactivating its normal function [7,53]. The results obtained in this study indicate that a similar C_60_ action may also affect SOD activity.

Some differences in the action of C_60_ and DOX on non-cancerous cells and cancer cells were also observed. Pre-treatment of DOX by fullerenol reduced or totally prevented the appearance of DOX toxicity in kidneys and testes of healthy male Wistar rats [54]. Research carried out by Prylutska et al. showed that the administration of C_60_ had no effect on the activity of SOD in non-cancerous liver and heart cells in mice. Prolonged DOX administration resulted in a decrease of SOD activity in both the liver and heart in mice [9]. Another possible explanation for the lower SOD activity in cells treated with the 50 mg/mL C_60_-2 μM DOX complex is the antioxidant action of C_60_ [25]. The amount of ROS, reduced by C_60_ and produced by DOX in the cells under investigation, could reduce the oxidative stress level in the cell and, consequently, reduce the activity of SOD. This type of C_60_ action was proposed by Prylutska et al., who demonstrated that complexing DOX with C_60_ leads to lower cytotoxicity of DOX to non-cancerous cells [9]. For this reason, the lower activity of SOD with higher concentration in cells treated with C_60_–DOX complexes observed in the experiment is particularly important in the context of using C_60_ as transport systems for DOX. Potential blocking of SOD activity in cancer cells would be a great advantage of C_60_ as transporters and would have a significant effect on enhancing the cytotoxic action of DOX.

Increased activity of SOD1 in breast cancer cells, compared with normal mammary cells, can have its contribution in the increased resistance of breast cancer cells to oxidative stress [55]. The inhibition of SOD activity by C_60_ can block the chemoresistance of cells caused by increased SOD activity that is also caused by DOX action. According to some reports, the inhibition of SOD1 leads to a decrease in the concentration of antiapoptotic factors, and finally to the apoptosis of lung cancer cells [56]. These findings indicate that the SOD1 isoform may play a key role in the survival mechanisms of tumor cells associated with oxidative stress. This makes SOD1 a potential target for anti-cancer therapies, and its inhibitors may find application in anticancer therapy.

The use of C_60_′s ability to accumulate in tumors and the ability to block MT concentration and SOD activity, shown in the current work, can be a great advantage of complexing anthracycline antibiotics with C_60_ and would have a significant effect on enhancing the cytotoxic action of DOX. At the same time, it is important to carry out further studies showing the effect of the used complexes on MT concentration and SOD activity in cardiomyocytes and liver cells that are the most exposed to DOX toxicity.

## 4. Materials and Methods

### 4.1. Chemicals

Lyophilized bovine SOD1 (Cat. no: S9697-15KU), doxorubicin HCl (Cat. no: D1515), fullerene (Cat. no: 379646), sodium bicarbonate (Cat. no: S 6014-1KG), sulphuric acid solution H_2_SO_4_ (Cat. no: 339741) and other chemicals were purchased from Sigma-Aldrich (St. Louis, MO, USA), unless noted otherwise. Nitric acid solution HNO_3_ from Lachner (Cat. no: 10023-ATO) was used. Sodium carbonate (Cat. no: 1331-11-810360-2) was obtained from POCH. EDTA (Cat. no: 11282) and *L*-epinephrine (Cat. no: 10980) were obtained from Serva. Hydrochloric acid was obtained from ChemPur (Cat. no: WE 231-595-7).

### 4.2. Preparation of Fullerene-Doxorubicin Complexes

Complexes of fullerene (C_60_) with DOX were prepared according to previous protocol [11]. Briefly, C_60_ (12.5 or 25 mg) in 4 mL of HNO_3_:H_2_SO_4_ (1:3) mixture was dispersed and heated for 7 h at 70 °C. Then, the suspension was put in ultrasonic bath for 30 min and centrifuged at 14,000× *g* for 30 min. The obtained sediment was washed with 500 μL H_2_O (18 MΩ) and centrifuged at 14,000× *g* for 30 min. The last step was repeated 3 more times. After the last centrifugation, the sediment was dispersed in 500 μL of 1.5 or 2.5 μM doxorubicin (DOX) solution, placed in ultrasonic bath for 90 min and centrifuged at 14,000× *g* for 30 min. The supernatant was removed; 500 μL of 1% acetic acid was added and put in ultrasonic bath for 30 min. The obtained sediment (C_60_–DOX complexes) was washed twice with 500 μL H_2_O (18 MΩ).

The entrapment efficiency (EE) of C_60_ was expressed as a DOX amount in fullerene juxtaposed with the initial amount of DOX used. The encapsulated DOX concentration was calculated from the calibration curve of the fluorescence signal. EE (%) was calculated using the following equation:

EE (%) = amount of DOX in fullerene/amount of initial DOX used × 100(1)

### 4.3. Scanning Electron Microscopy

The morphology of complexes was assessed using scanning electron microscopy as stated before [11]. Experiments were conducted by depositing 10 μL of aqueous solutions of the fullerene and C_60_–DOX complexes on a freshly cleaved mica grid and allowing them to dry for 60 min in air. A thin film of Au was then sputtered onto the samples. The samples were imaged with a FEI NOVA Nano400 scanning electron microscope. Accelerating voltage of 15 kV and beam currents of about 1 nA were used.

### 4.4. Dynamic Light Scattering

The size distribution was determined by dynamic light scattering using a Zetasizer Nano ZS ZEN3600 (Malvern Instruments, Worcestershire‎, UK) with a detection angle of 173° in optically homogeneous square polystyrene cells. The samples were diluted hundredfold with deionized water. All measurements were performed at 25 °C. Each value was obtained as an average of five runs with at least 10 measurements. Version 7.10 of the Zetasizer software was applied for data evaluation.

The particle charge (ζ-potential) was measured by the microelectrophoretic method using a Malvern Zetasizer Nano ZS ZEN3600 (Malvern Instruments). All the measurements were performed at 25 °C in polycarbonate cuvettes. Each value was obtained as an average of three subsequent runs of the instrument with at least 10 measurements.

### 4.5. Fluorescence Measurements of DOX

Fluorescence scans of DOX alone and in complexes with C_60_ were carried out using a multifunctional microplate reader (Infinite M200, TECAN, Zürich, Switzerland). The measurement conditions were as follows: excitation wavelength—480 nm, emission wavelength ranging from 510 to 700 nm, gain—180, number of flashes—35, integrated time—50 µs. The samples (100 µL) were placed in a 96-well UV plate (Imaging Plate 96-FC TC-Surface, Zell-Kontakt GmbH (Cat. no: 3242-20), Northeim, Germany). The calibration curve of DOX was determined with free DOX concentrations of 0, 8, 16, 33, 66, 131, 263, 525, 1050 and 2100 nM, and the measured DOX concentrations were 1 and 2 µM in the prepared C_60_–DOX complexes.

### 4.6. Growth Measurement

MCF-7 cells, a human breast cancer cell line obtained from the American Type Culture Collection (Rockville, MD, USA), were cultured until passage 30. The MCF-7 cells were maintained as monolayers in RPMI 1640 medium supplemented with 2 g/L sodium pyruvate, 1.2 mM glutamine (pH 7.4), 10% (*v*/*v*) fetal bovine serum, 100 U/mL penicillin and 100 U/mL streptomycin at 37 °C in a humidified environment containing 5% CO_2_.

Real-time analysis of proliferation was performed using the xCELLigence system (RTCA DP, ACEA Biosciences Inc., San Diego, CA, USA). The cells were grown in a disposable E-Plate 16, a microtiter plate with gold electrode structures of biocompatible surface at the bottom of each well. The electrode and its respective counter electrode have a complex interdigitated structure and both combined cover 80% of the ground. The RTCA device measures the impedance at an alternating voltage of 20 mV and 10 kHz frequency. As the whole device is housed inside the incubator, the cells are kept under optimal culture conditions (5% CO_2_, 37 °C) during the measurement. After seeding (80,000 cells/well-200 µL) the cell suspensions into the wells, the cells were left to grow. A day after seeding, the medium was changed and the cells were treated with 10 μL of different concentrations of DOX, C_60_ and C_60_–DOX complexes mixtures; their growth was monitored for 70 h.

### 4.7. Cell lysis and Total Protein Concentration Analysis

Cell lysis was performed with a Passive Lysis 5× Buffer (Promega, Cat. no: E1941). 50 μL of the buffer and 150 μL of distilled water was added to 50 μL of the cell sample to obtain a fivefold dilution of the buffer. The lysis was kept under stirring for 20 min, after which the samples were centrifuged and the supernatant was removed.

Total protein concentration was measured by the Bradford method. A total of 20 µL of a sample was added to 200 µL of reagent (0.01% Coomassie brilliant blue G-250, 4.7% ethanol, 8.5% phosphoric acid in distilled water). Detection was carried out at 590 nm within a 15-min time period.

### 4.8. Metallothionein Concentration

Concentration of MT-1/2 in the cell lysate was measured following the procedure described by Milnerowicz and Bizoń; a two-step direct enzyme-linked immunosorbent assay (ELISA) [57] with modification of the primary antibodies. A primary commercial monoclonal antibody, UC1MT (Cat. no.: MA1-25479, ThermoFisher) was used in 1:5000 dilution. This antibody detects both MT-1 and MT-2 isoforms and the product detected is referred to as MT-1/2 in this report. As indicated in the above-mentioned publication, a secondary biotinylated polyclonal goat anti-mouse IgG antibody (Cat. no: E0433, DakoCytomation) and horseradish peroxidase (HRP)-avidin (Cat. no.: P0347, DakoCytomation) were applied in 1:400 and 1:8000 dilution, respectively. The reaction was visualized by ortophenylenediamine in 0.25 M phosphate citrate buffer (citric acid, Cat. no.: 251275, Sigma-Aldrich; sodium phosphate dibasic, Cat. no.: S7907, Sigma-Aldrich), pH 5.5 containing 0.006% H_2_O_2_. The color reaction was stopped using 50 µL of 3 M HCl. Absorbance was measured at 490 nm and 630 nm as the reference wavelength. MT concentrations in the cell lysate were converted to the amount of protein measured by the Bradford method [58].

### 4.9. Concentration of SOD1 and Cu/Zn SOD Activity

SOD1 concentration was determined by ELISA (ThermoFisher, Cat. No. BMS222) and converted to the total protein concentration measured by the Bradford method. Briefly, human Cu/Zn SOD present in the sample and human SOD1 standards bound to anti-human Cu/Zn SOD coating antibodies adsorbed to the microwells. An HRP-conjugated anti-human Cu/Zn SOD antibody was added and bound to human Cu/Zn SOD captured by the first antibody. Following incubation, unbound HRP-conjugated anti-human Cu/Zn SOD was removed during the wash step, and tetramethylbenzidine was added to the wells as a substrate solution reactive with HRP. A colored product was formed in proportion to the amount of human Cu/Zn SOD present in the sample or standard. The reaction was terminated by adding 1 M phosphoric acid and absorbance was measured at 450 and 620 nm as the reference wavelength. A standard curve was prepared using human Cu/Zn SOD standard dilutions.

Cu/Zn SOD activity was assayed with the use of the epinephrine method [59]. The assay was held in 30 °C, in 50 mM carbonate buffer, with 100 µM of EDTA. *L*-epinephrine (10 mM) was dissolved in 10 mM hydrochloric acid. Epinephrine, upon exposition to alkaline pH (10.2), is autooxidized to adrenochrome. The product was determined by absorption spectroscopy at 480 nm using Specord 40 spectrophotometer (Analytik Jena, Jena, Germany). In the presence of SOD, the rate of epinephrine autooxidation is decreased due to the ongoing dismutation process. One unit of SOD activity is equal to a 50% decrease in the rate of adrenochrome formation.

### 4.10. Statistical Analysis

All the experiments were performed in triplicate. Statistical analysis was done using the Statistica 9.1 (StatSoft, Tulsa, OK, USA) software with the acquisition of mean values and standard deviation. The normality of the variables was analyzed using the Shapiro–Wilk W test. Student’s *t*-test was used to evaluate the significance of the differences between groups. The significance level was established at *p* ≥ 0.05.

## 5. Conclusions

The complexation of DOX with C_60_ increases the cytotoxicity of the drug toward the evaluated tumor cell line. Treatment with the C_60_–DOX complex resulted in an increase in the inhibition of cell proliferation compared to that by DOX alone.

The obtained results suggest a great prospect of applying C_60_–DOX complexes in the chemotherapy of malignant tumors. MT and SOD can be considered as a novel target for cancer therapy. By leading to an elevated ROS level, deregulation of the antioxidant machinery appears to play a critical role during the transformation.

## Figures and Tables

**Table 1:** Activity of SOD in conversion to SOD1 concentration in MCF-7 cells treated by DOX, C_60_ and C_60_–DOX complexes.

DOX/C_60_	SOD1 Concentration (µg/mg of Total Protein)	SOD Activity (U/mg of Total Protein)	SOD Activity/SOD1 Concentration (U/µg SOD1)
0	0.10	10.44	104.4
1 µM DOX	0.14	18.56	132.5
2 µM DOX	0.40	11.03	27.6
25 mg/mL C_60_	0.54	9.85	18.2
50 mg/mL C_60_	0.31	5.85	18.9
25 mg/mL C_60_ +1 µM DOX	0.50	16.23	32.5
50 mg/mL C_60_ +1 µM DOX	0.32	8.65	27.0
50 mg/mL C_60_+2 µM DOX	0.39	7.77	19.9

## Abbreviations

C_60_	fullerene
DOX	doxorubicin
EE	entrapment efficiency
MT	metallothionein
ROS	reactive oxygen species
SOD	superoxide dismutase
